# Electronic monitoring of self-reported mood: the return of the subjective?

**DOI:** 10.1186/s40345-016-0069-x

**Published:** 2016-11-29

**Authors:** Abigail Ortiz, Paul Grof

**Affiliations:** 1 Department of Psychiatry, University of Ottawa, Ottawa, ON Canada; 2 Mood Disorders Centre of Ottawa, University of Ottawa Health Services, 100 Marie Curie Private, Suite 300, Ottawa, ON K1N 6N5 Canada; 3 Department of Psychiatry, University of Toronto, Toronto, ON Canada

**Keywords:** Mood disorders, Apps, Episode prediction, Youth, Mood stabilizers, Treatment

## Abstract

This narrative review describes recent developments in the use of technology for utilizing the self-monitoring of mood, provides some relevant background, and suggests some promising directions. Subjective experience of mood is one of the valuable sources of information about the state of an integrated mind/brain system. During the past century, psychiatry and psychology moved away from subjectivity, emphasizing external observation, precise measurement, and laboratory techniques. This shift, however, provided only a limited improvement in the understanding of mood disorders, and it appears that self-monitoring of mood has the potential to enrich our knowledge, particularly when combined with the advances in technology. Modern technology, with its ability to transfer information from the individual directly to the researcher via electronic applications, enables us now to study mood regulation more thoroughly. Frequent subjective ratings can be helpful in identifying individualized treatment with effective mood stabilizers and recognizing subtypes of mood disorders. The variability of subjective ratings may also help us estimate the increased risk of recurrence and guide a tailored treatment.

## Background

Subjective experience of mood is one of the valuable sources of information about the state of the mind/brain function. While building the foundations of psychiatry during the nineteenth century, clinicians paid considerable attention to the subjective feelings of patients. Kraepelin, for instance, put emphasis on the subjective experience of patients when describing diagnostic entities (Kraepelin and Diefendorf 1902).

The twentieth century psychiatry and psychology attempted to move away from the subjective experience, questioning their validity, especially under the influence of behaviorism. Researchers tended to neglect subjective experience. Introspection was considered mostly unreliable and scientifically unsatisfactory. Psychologists in particular long viewed self-reports with marked suspicion (Clark and Watson 1991). In addition, there was a tendency to accept self-reports only if some physiological measurements authenticated them.

Researchers in both fields emphasized external observation, laboratory techniques, and precise measurement. But despite a century limited predominantly to such objective strategies, the yield in terms of understanding the nature of mood disorders has been limited. Even after a century of neurobiological research, no single psychobiological measurement has emerged that expresses the activity of these complex regulations in an integrated way altogether as well as the subjective experience of the individual can do. While the search for the biological basis of mood disorders continues and expands to new areas, there has recently been some renaissance of interest in better utilizing the subjective experiences of the patients.

With the emergence of mood stabilizers, and when behaviorism gradually declined as a leading force, a new paradigm started to take place, in which mood—the color of the subjective experience—is seen as a rich source of information with a rather complex regulation. Mood regulation can be conceptualized as a buffering system, allowing flexible responses that enable us to adapt to an ever-changing range of stimuli (Ortiz et al. 2015). Given the complex control of many brain functions, subjective experience of mood as a single expression of integrated functions has a special value.

Emotions exert a profound influence on human life and human behavior, and markedly alter even major economic decisions people make (Lerner et al. 2015). The research interest in mood disorders expanded by recognizing the importance of the subjective experience and the individual’s moods, to enrich the information obtained from objective measurements.

## Psychological studies of mood ratings

Emotions are usually viewed as fundamental, distinctly subjective affective states of shorter duration, accompanied by bodily expressions and autonomic changes. Ordinarily, negative emotions (such as sadness, fear, anger, or disgust) comprise only a small fraction of everyday affective experience in controls. Moods are usually defined as affective states that may last from several hours to several days and are strongly influenced by external events by factors such as stress, social activity, and exercise (Powers et al. 2015), as well as from endogenous cycles or rhythms (Powers et al. 2015; Murray et al. 2009). It is mood, rather than emotions, that provides a better understanding of everyday experience.

Psychologists have been carefully investigating serial subjective assessments of mood states and have discovered interesting correlations (Larsen and Ketelaar 1991). David Watson, in particular, examined systematically short-term mood fluctuations and their relationship to temperament and synthesized a vast body of knowledge (Watson 2000). These observations have been generated mostly on cohorts without a diagnosed psychiatric illness, but can be considered extendable to many patients with mood disorders treated on an outpatient basis. We extract some of the conclusions that may be useful to psychiatric observations.

In psychological research it has been shown that the affective experiences of an individual can be subsumed under two general dimensions: negative and positive mood states (Watson 2000). However, these relevant concepts have not yet had much impact on psychiatric research of mood disorders. Negative mood states involve the experience of negative emotions and poor self-concept. It subsumes a variety of negative emotions including anger, contempt, disgust, guilt, and fear. Positive mood states, as a characteristic that describes how we experience positive emotions and interact with others, is characterized by enthusiasm, alertness, energetic, and active traits. These two dimensions are nearly independent of each other; it is possible for someone to be high in both positive and negative traits; or high in one and low in the other; or low in both (Watson 2000). These traits have been found to be moderately stable over time and across situations.

Within these two dimensions, distinct affective experiences are meaningfully intercorrelated. For instance, neuroticism and extraversion traits strongly correlate with individual differences in negative and positive emotional experience (Lyubomirsky et al. 2005). Negative affect, a highly non-specific dimension that is common to many types of psychopathology, correlates moderately with neuroticism. Negative mood states are much more responsive to ongoing stress and current life crises; in contrast, positive mood states are strongly associated with social interaction and physical activity (Watson 1988, 2000).

## Clinical applications

In clinical practice, the primary tasks are to identify the illness early and treat it effectively. To start with, self-monitoring of mood can be helpful in clinical practice when evaluating whether a given treatment is useful. In addition, daily ratings and their appropriate analysis, in combination with objective data, will allow, for example, to differentiate among offsprings of bipolar parents those who will stay well and those who have a high risk of developing a mood disorder; to select those patients who will be likely stabilized on lithium versus those who require neuroleptics or lamotrigine; and to identify time periods when a patient is at a high risk of recurrence (Ortiz et al. 2016).

### Identifying predisposed youth

Minor mood disorders (depression NOS, cyclothymia, dysthymia), along with childhood sleep and anxiety disorders precede and predict the onset of diagnosable major mood disorders (Grof et al. 2009; Duffy et al. 2013). Adolescents often experience subthreshold psychiatric symptoms—sadness, irritability, anxiety, elation, energy changes, and sleep problems—even years before a recognizable mood disorder (Duffy 2014). These changes cannot be reliably captured retrospectively and benefit from daily monitoring prospectively. One such prospective mood monitoring study that is underway is the True Colors study (Bonsall et al. 2012, 2015). This study uses mobile phone and web-based technology to collect self-reported health measures on a daily and weekly basis. This monitoring has been well accepted by patients, particularly the younger age group who are increasingly familiar with widespread mobile devices and social media. The investigators expect to find a significant difference in mood stability profiles between high-risk offspring and controls. Further, in those high-risk offspring with evidence of mood instability, they expect a higher risk of new onsets of diagnosable mood episodes over the two years of study. A similar, cross-sectional study suggests that mood instability may be a prodromal phenotype for BD in the offspring of parents with bipolar disorder compared to controls (Birmaher et al. 2013; Howes et al. 2011). If young people who are at high risk of developing serious and persistent mood disorders could be identified earlier this would enable them to receive appropriate, timely treatment, and reduce the associated morbidity and mortality, given that the risks of complicating addictions, school drop-out, and suicide are all greater early in the illness.

### Contributing to the choice of an effective stabilizer

**Fig. 1:**
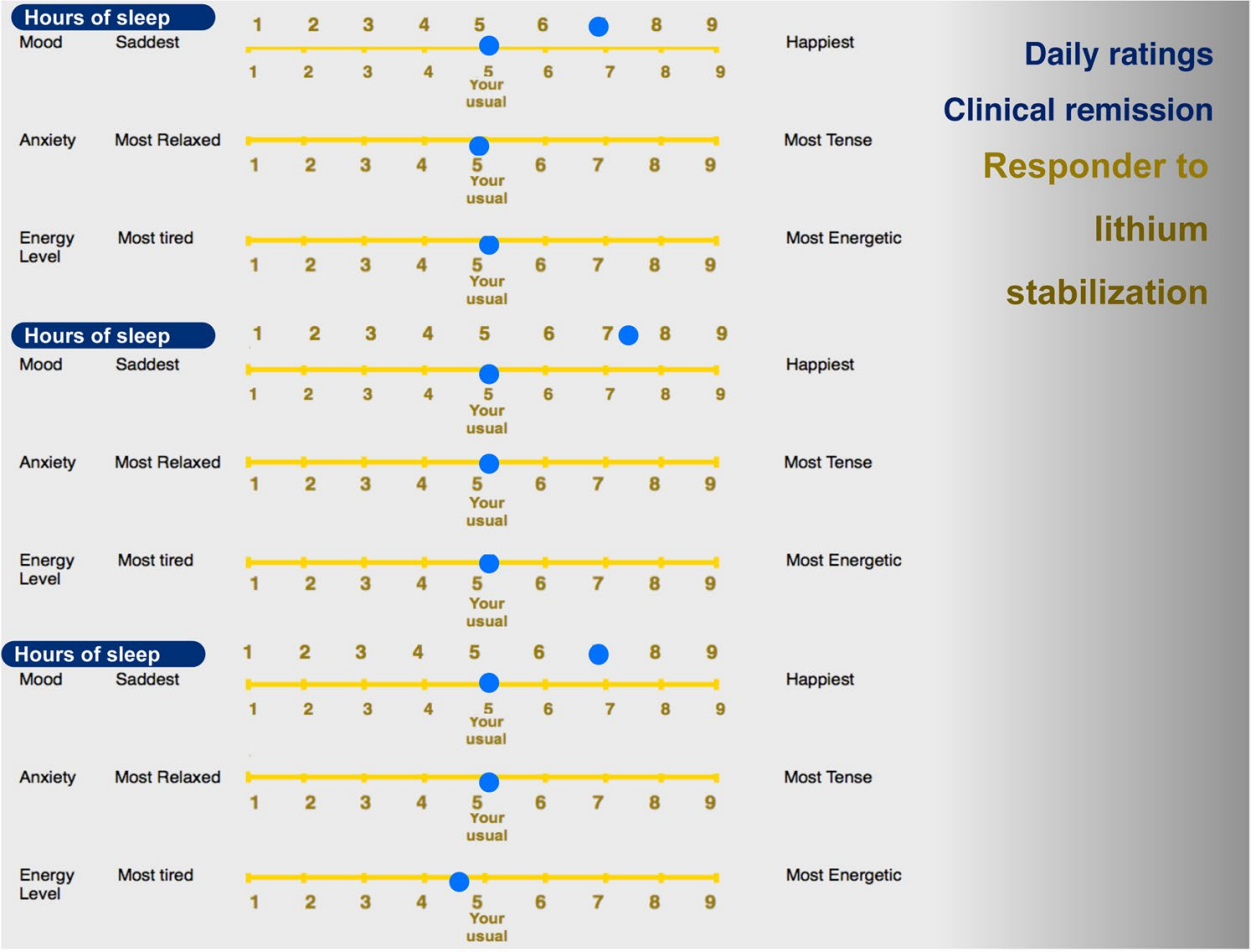
Mood ratings in clinical remission for lithium responders. When in remission, lithium responders mark their mood right in the middle (the point that the patient considers where his/her normal mood is), experiencing distressing symptoms very rarely. On the other hand, in patients with bipolar spectrum disease who fail to respond to lithium and require stabilization with neuroleptics (Fig. 2) or lamotrigine (Fig. 3), some deviation from the midline is usually present most of the time

**Fig. 2:**
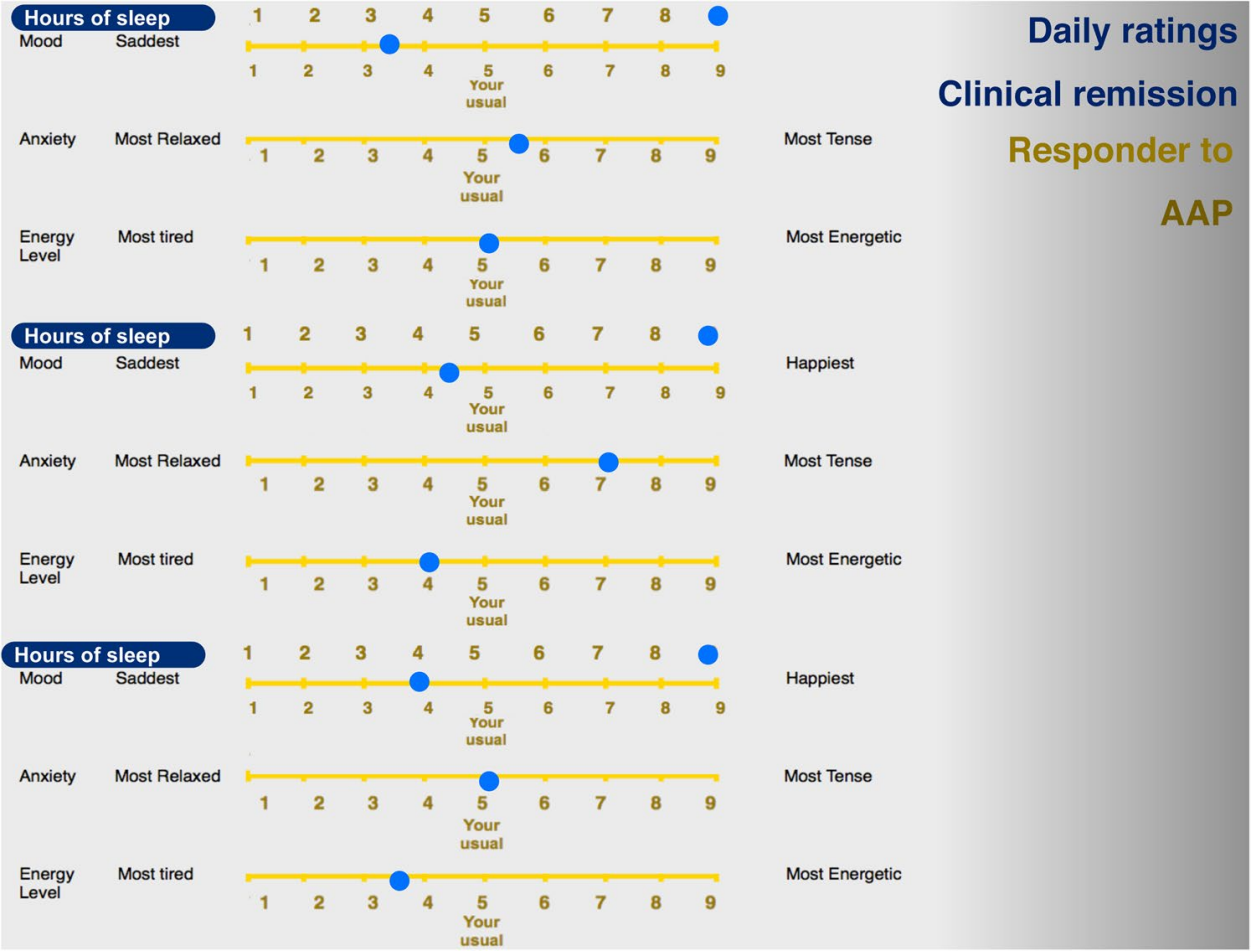
Mood ratings in clinical remission for responders to atypical antipsychotics (AAP). In these patients, even when in clinical remission, some deviation from the midline (the point that the patient considers where his/her normal mood is) is usually present most of the time

**Fig. 3:**
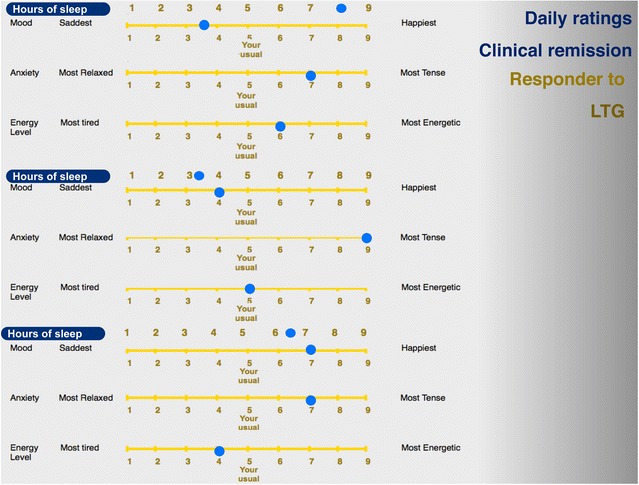
Mood ratings in clinical remission for responders to lamotrigine (LTG). When in remission, patients who benefit most from lamotrigine often find it difficult to express their daily mood by marking a single point. Their symptoms frequently change several times a day, and would need several points in a day to capture the changes. In addition, these patients often recognize life events and external circumstances as triggers for significant changes in their mood

Identifying the type of illness and tailoring of a mood stabilizer to the individual clinical profile of a patient with bipolar disorder is an example of the utility of daily ratings in clinical practice (Grof 2003). It must be stressed that the information critical for the choice of an effective stabilizer comes first among the other aspects of clinical profile, such as family history, the type of clinical course (episodic or non-episodic) or comorbidity (Grof et al. 1994). The observations described in this section about daily self-report of mood, anxiety, energy, and sleep were obtained from over one thousand patients participating in our clinical research programs. As part of the initial assessment, every new patient has had to provide a minimum of six weeks data to help with the diagnostic assessment. The relationship with the treatment outcome was investigated in retrospect. Our observations show that patients with the classical type of bipolar disorder and with episodic, recurrent depressions who can be stabilized very well with lithium salts, show characteristic patterns of daily ratings. Both patients and their clinicians can usually distinguish between the time when the patient is in full remission and when the patient relapses (Figs. 1, 2, 3): in remission, patients keep marking their mood right in the middle (the point that the patient considers where his/her normal mood is), experiencing distressing symptoms very rarely. On the contrary, when ill, their symptoms keep changing hand-in-hand; i.e., during a depressive episode the experience of low mood is most of the time associated with the experience of low energy and elevated anxiety.

On the other hand, patients with bipolar spectrum disease who fail to respond to lithium and require stabilization with neuroleptics or lamotrigine, rarely show days fully free of symptoms for any length of time. Some deviation from the midline is usually present most of the time.

Furthermore, the patients who benefit most from lamotrigine often find it difficult to express their daily mood by marking a single point. Their symptoms frequently show “ultra-rapid” cycling, changing several times a day, and would need several points in a day to capture the changes. In addition, these patients very often recognize and comment on life events and external circumstances triggering significant changes in their mood.

Double-blind clinical trials comparing the three groups of stabilizers head-to-head and identifying responders have not been carried out and are unlike to take place, because of the cost and lack of interest. Fortunately, we can utilize systematic clinical observations of response as they markedly improve clinical practice.

Another interesting approach, similar to the one we are currently undertaking, includes the analysis of early response of mood instability to mood stabilizers as a new test paradigm that incorporates daily ratings and non-linear analyses (Harrison et al. 2016; Holmes et al. 2011).

### Forecasting risk of recurrences in mood disorders

Mood variability patterns are different among patients with different diagnoses (Grof et al. 1994; Katerndahl et al. 2007). Several studies have shown that high levels of mood regularity (low variability, in other words) are characteristic of disease states particularly in patients with major depression (Katerndahl and Wang 2007), posttraumatic stress disorder (Cowdry et al. 1991), or panic disorder (Katerndahl and Wang 2007).

In bipolar disorder, up until recently, the emphasis has been placed on acute episodes of depression or mania, underestimating the subtleties of mood changes in-between episodes as a key feature of the disorder. We have shown different patterns of mood variability between healthy controls and euthymic bipolar patients in our previous studies: essentially, healthy controls showed higher mood variability levels, whereas stable bipolar patients showed low mood variability, in keeping with a less flexible system (Ortiz et al. 2015). These data suggest that the variability of subjective ratings may help us estimate the increased risk of recurrence and guide an effective, individually tailored treatment. A new protocol (Ortiz et al. 2016), currently underway, aims to use non-linear techniques to study mood variability in patients with mood disorders to predict high-risk times for the onset of episodes.

Other studies, also in bipolar disorder, have used a combination of actigraphy and self-assessment questionnaires a combination of actigraphy and self-assessment questionnaires sent by text messaging (Novak et al. 2014). Although it is unclear whether these self-assessment questions were previously validated, this is another example of the utility of subjective measurements and remote monitoring to establish whether a patient is at risk for relapse.

This type of approach could be related to better clinical monitoring, aimed at intermittent treatment, with few side effects and optimal recovery. Moreover, this approach can be particularly valuable to adolescents at risk—either because of a strong family history or previous episodes and because mobile technology and social media are part of their lives.

However, one important limitation of using self-ratings and self-monitoring is the severity of mood disorder. Neither manic nor severely depressed patients are capable of providing valid self-ratings of their mood states.

## Electronic mood monitoring and utilization of apps

One of the first efforts to include a more comprehensive, electronic self-mood monitoring, medication compliance, and sleep was Chronorecord (Bauer et al. 2005, 2008; 2012; Whybrow et al. 2003). Using this approach in large, international studies, the authors have productively analyzed many aspects of bipolar disorder, ranging from clinical course (Bauer et al. 2006, 2007, 2010, 2011a, b, 2012; Rasgon et al. 2005), treatment adherence (Bauer et al. 2010, 2013a, b, c; Adli et al. 2005), sleep (Bauer et al. 2006, 2008, 2009), among others (Conell et al. 2016; Bauer et al. 2005, 2006, 2009b, c; Glenn et al. 2006). Other efforts have included the use of life charts by the Stanley Foundation Network (Leverich et al. 2001). More recently, as previously mentioned, the Oxford group has also developed the “True Colors” system, which also monitors mood remotely (Holmes et al. 2011; Miklowitz et al. 2012; Moore et al. 2014). True Colors provides a way of visualizing mood data over time. The data are made available to the patient, which allows the patient to be more aware of their mood variability, activity, and sleep. It allows them to monitor how much they are helped by a new medication or a particular psychological treatment. At the same time, creating an automatic system that picks up early signs of deterioration from self-reports is quite advantageous and will allow to study the specific effects of a drug. In this particular case, the OxLith trial (Saunders et al. 2016), is a high intensity RCT which seeks to discover new insights into lithium’s mechanism of action and eventually generate new leads for drug discovery.

Modern technology with its ability to transfer information from the individual directly to the researcher via electronic applications (“apps”) has moved the investigations further ahead. They make it relatively easy to collect the subjective rating of symptoms daily and submit them directly to automated analysis so that warning signs can then be communicated directly to the treatment team. Over 165,000 health apps are now directly available to patients; mental health is the largest group of apps for a specific disease state, larger than cardiology, endocrine, or other disorders (Torous et al. 2016). Patients are already bringing apps, sleep-tracking devices and activity monitoring devices to psychiatrists to share their data; however, research is needed to determine consistent means for evaluating the performance of apps. Currently, as a result of the dearth of information on regulatory practices for apps, several authors have developed standardized frameworks for their clinical use (BinDhim et al. 2015; Lewis and Wyatt 2014; Donker et al. 2013).

Mental health apps have the potential to be useful tools to complement clinical practice, but the majority of those currently available lack scientific evidence about their efficacy (Marley and Farooq 2015). In addition, they have a few challenges, including concerns about how the data will be used by the app service, lack of medical involvement in app development, patient confidentiality issues, clinical risk emerging from the use of apps, and lack of evidence-based practice recommendations. Moreover, there are societal and ethical implications related to the utilization of these for both medical and non-medical purposes (Glenn and Monteith 2014).

Researchers from the Copenhagen Affective Disorders Research Center developed a smartphone-based system which included daily subjective assessments of activity in BD patients, as well as a feedback loop between patients and clinicians. The system proved to be highly useful by patients, with high rates of self-assessment adherence (Bardram et al. 2013). The authors found that the severity of depression and mania ratings correlated with smartphone-generated data, including physical activity (Faurholt-Jepsen et al. 2014) and voice features (Faurholt-Jepsen et al. 2016). However, mood monitoring combined with a feedback loop system to clinicians did not result in decreased depressive or manic symptoms (Faurholt-Jepsen et al. 2014).

These findings are in keeping with the fact that mobile technology is well suited for augmenting the capability of the psychiatrist to deliver high-quality care (Hsin et al. 2016), and that both sides of the equation are needed in order for this enterprise to be successful (Spaniel et al. 2015). For this augmentation to work well, the apps should be embedded in clinical care, developed in close partnership with clinicians and patients, and combined with those aspects of the system that we know already work well.

Technology can support and enhance clinical practice (Torous and Baker 2016), for example, by incorporating data from the app and physiological data into electronic medical records. This method can allow a prompt detection of mood episodes (Grof et al. 1993), and possibly allow intermittent treatment, with lithium salts, for example, in those patients with a classical type of bipolar illness, which will decrease side effects and improve compliance. In the long term, these approaches could decrease rates of admission and suicide and will overall improve the quality of life of patients with mood disorders.

In this context, apps are becoming a technological advance enabling us to study mood regulation and to treat mood disorders better and more thoroughly.

## Conclusions

Subjective experience of mood is one of the valuable sources of information about the state of an integrated mind/brain system. During the past century, psychiatry and psychology moved away from subjectivity, emphasizing external observation, precise measurement, and laboratory techniques. This shift, however, provided only a limited improvement in the understanding of mood disorders, and it appears that self-monitoring of mood has the potential to enrich our knowledge, particularly when combined with the advances in technology.

We have described three examples in which frequent monitoring of subjective mood ratings are being utilized in an attempt to identify the offsprings of bipolar parents at high risk of becoming later ill, to forecast the time of future episodes of illness and to select the optimal mood stabilizer for the patient. With the advantages of modern technologies, adopting an approach that combines subjective mood ratings and objective data will allow us to estimate the risk of recurrence and to guide an effective and tailored treatment for patients with mood disorders.
